# Supplementary material for: Long Non-Coding RNAs Might Regulate Phenotypic Switch of Vascular Smooth Muscle Cells Acting as ceRNA: Implications for In-Stent Restenosis
Source: Int J Mol Sci. 2022 Mar 12;23(6):3074. doi: 10.3390/ijms23063074 (PMC8952224; doi:10.3390/ijms23063074)
Supplement: Supplementary file 1 [file ijms-23-03074-s001.zip › ijms-1623169-supplementary/Supplementary Figure S1.pdf]

A

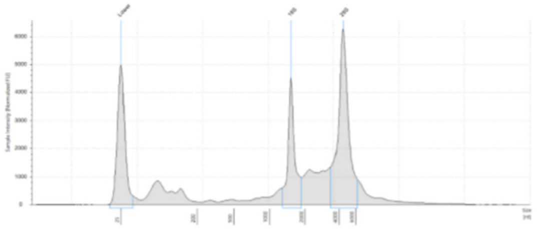

B

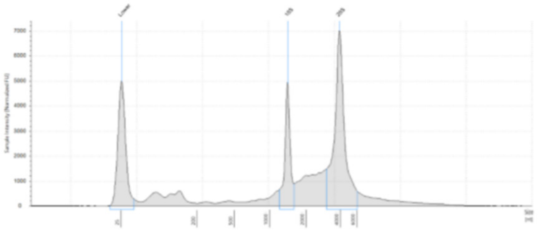

C

Sample	Conc (ng/µL)	Vol (µL)	Amount (µg)	RIN	28S/18S	OD260/280
Cont_1	69,5	50	3475	9,6	2,2	1,89
Cont_2	18,8	50	940	9,4	2,4	1,84
Cont_3	35,6	50	1780	9,4	2,7	1,93
Stent_1	33,4	50	1670	9,8	2,5	1,87
Stent_2	35,2	50	1760	9,7	2,7	2,03
Stent_3	36,5	50	1825	9,6	2,5	2,16

Supplementary Figure S1. RNA extraction quality control. RNA integrity was assessed by Agilent 4200. Panel **A** shows Cont_1 and panel **B** shows Stent_1 RNA analysis as a representative sample for each group. Panel **C** describes the characteristics of all samples. RIN, RNA integrity number, Cont_ contractile vascular smooth muscle cells, Stent_ Stent induced proliferative phenotype cells, OD optic density.
