# Supplementary material for: Long Non-Coding RNAs Might Regulate Phenotypic Switch of Vascular Smooth Muscle Cells Acting as ceRNA: Implications for In-Stent Restenosis
Source: Int J Mol Sci. 2022 Mar 12;23(6):3074. doi: 10.3390/ijms23063074 (PMC8952224; doi:10.3390/ijms23063074)
Supplement: Supplementary file 1 [file ijms-23-03074-s001.zip › ijms-1623169-supplementary/Supplementary Figure S2.pdf]

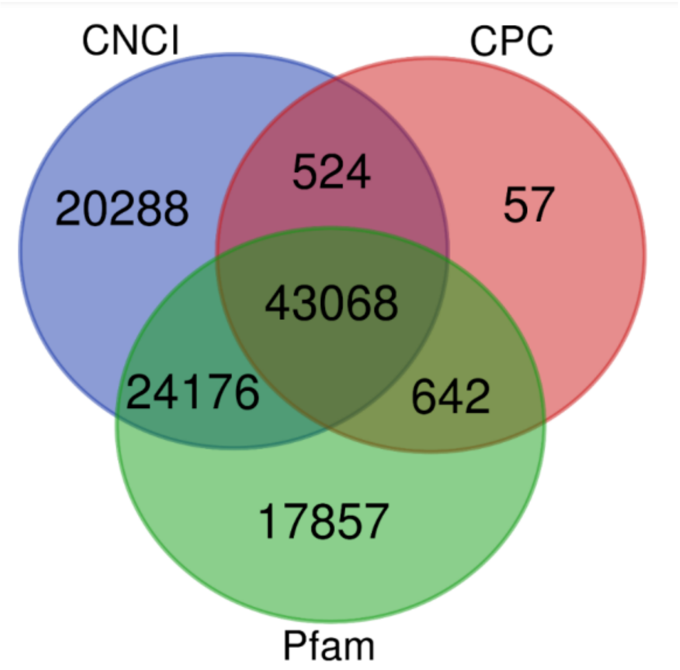

Supplementary Figure S2. Coding potential analysis of transcripts. Venn diagram of results from three predictive tools. Number in each circle and overlap represent the respective total and shared number of noncoding transcripts predicted by the software.
