# Supplementary material for: Long Non-Coding RNAs Might Regulate Phenotypic Switch of Vascular Smooth Muscle Cells Acting as ceRNA: Implications for In-Stent Restenosis
Source: Int J Mol Sci. 2022 Mar 12;23(6):3074. doi: 10.3390/ijms23063074 (PMC8952224; doi:10.3390/ijms23063074)
Supplement: Supplementary file 1 [file ijms-23-03074-s001.zip › ijms-1623169-supplementary/Supplementary Table S1.pdf]

Supplementary Table S1. Summary of mapping results for each sample

Sample ID	Total read	Reads mapped	Unique mapped	Multi mapped
Cont_1	141467314	94.5 %	71.2 %	23.3 %
Cont_2	138907016	84.4 %	50.0 %	34.4 %
Cont_3	146801934	92.3 %	66.9 %	25.7 %
Stent_1	132026982	84.1 %	67.2 %	16.9 %
Stent_2	116954322	83.9 %	66.9 %	17.0 %
Stent_3	110719626	84.2 %	67.6 %	16.6 %

Cont_ Contractile Human umbilical artery smooth muscle cells, Stent_ Stent induced proliferative phenotype
